# Acute effects of combined isometric and plyometric conditioning activities on sports performance and tendon stiffness in female volleyball players

**DOI:** 10.3389/fphys.2022.1025839

**Published:** 2022-10-11

**Authors:** Rafał Kalinowski, Anna Pisz, Dominik Kolinger, Michał Wilk, Petr Stastny, Michał Krzysztofik

**Affiliations:** ^1^ Department of Exercise and Sport Performance, The Jerzy Kukuczka Academy of Physical Education in Katowice, Katowice, Poland; ^2^ Faculty of Physical Education and Sport, Charles University, Prague, Czechia; ^3^ Institute of Sport Sciences, The Jerzy Kukuczka Academy of Physical Education in Katowice, Katowice, Poland

**Keywords:** post-activation performance enhancement, post-activation potentiation, athletic performance, resistance training, skin surface temperature, myotonometry, change of direction, countermovement jump (CMJ)

## Abstract

This study aimed to compare the effects of bilateral and unilateral conditioning activities (CA; combined isometric and plyometric) on countermovement jump performance, modified t-agility test, Achilles tendon stiffness and skin surface temperature. Thirteen female semi-professional volleyball players performed two CAs in random order: 1) bilateral isometric half back squats followed by bilateral drop jumps (BI-CA); and 2) unilateral isometric half back squats followed by unilateral drop jumps (UNI-CA). To assess the effects of CAs, countermovement jump, modified t-agility test, Achilles tendon stiffness and skin surface temperature measurements were performed 5 min before and 6 min after the CA. Both CAs significantly increased thigh skin surface temperature from pre- to post-CA (BI-CA, *p* < 0.001; effect size [ES] = 1.41 and UNI-CA, *p* = 0.001; ES = 1.39) but none of them influenced modified t-agility test time (interaction: *p* = 0.338, main effect of time: *p* = 0.121 and condition: *p* = 0.819). The countermovement jump height and modified reactive strength index significantly increased from pre-to post-CA during the BI-CA condition (*p* = 0.003, ES = 0.45, and *p* = 0.008, ES = 0.48) but not for UNI-CA (*p* = 0.061, ES = 0.18 and *p* = 0.065, ES = 0.26). No significant impact has been found for countermovement depth (interaction: *p* = 0.054, main effect of time: 0.097, and condition: *p* = 0.41) as well as for contraction time (interaction: *p* = 0.536, main effect of time: *p* = 0.224, and condition: *p* = 0.807). Moreover, stronger and weaker limb CMJ relative peak force significantly decreased from pre-to post-CA (*p* = 0.014, ES = −0.31, and *p* = 0.027, ES = −0.26; respectively) during UNI-CA condition but not for BI-CA (*p* = 0.096, ES = 0.23, and *p* = 1.41, ES = 0.18). The stronger and weaker limb Achilles tendon stiffness significantly increased from pre-to post-CA during the UNI-CA condition (*p* = 0.013, ES = 0.60 and *p* < 0.001, ES = 0.79; respectively) but not for BI-CA (*p* = 0.66; ES = 0.15 and *p* = 0.265; ES = 0.42). Furthermore, the post-CA stronger limb Achilles tendon stiffness during the UNI-CA was significantly higher than that noted during the BI-CA (*p* = 0.006, ES = 0.7). The present study showed that combined isometric and plyometric bilateral CA effectively improved the countermovement jump but did not enhance the t-agility test performance. These findings indicate that exercise combinations could effectively produce a post-activation performance enhancement effect but should replicate the following explosive task as much as possible.

## Introduction

One of the training methods used to enhance explosive performance acutely is applying specific conditioning activity (CA) prior to a similar movement task (Seitz and Haff, 2016). Typically, a CA consists of a high-intensity isotonic or isometric exercise performed before an explosive exercise with a similar movement structure, for instance, high-loaded back squats performed before the vertical jumping (Seitz and Haff, 2016). Generally, this performance improvement is called a post-activation performance enhancement (PAPE) effect and is most often registered 6–10 min after completing the CA (Seitz and Haff, 2016; Krzysztofik et al., 2021).

The physiological mechanisms underlying the PAPE effect are probably associated with changes commonly observed during the warm-up, such as increased muscle temperature, fiber water content, and muscle excitation (Blazevich and Babault, 2019). Referring to changes in muscle temperature, a safe, quick, and effective method might be infrared thermography which allows examining of the changes in skin surface temperature (Costello et al., 2013). Infrared thermography can be used to measure changes in temperature caused by heat generation induced by muscle contraction during physical activity (Chudecka, 2013). Although, to the authors’ knowledge, the changes in skin surface temperature have not been evaluated directly in studies devoted to the PAPE phenomena. It is known that an increase in muscle temperature might contribute to neuromuscular performance improvements (Racinais et al., 2017). However, in PAPE studies, performance improvements are reported also after very low-volume CA, which does not appear to cause a significant increase in muscle temperature. For example, in a study by Tsoukos et al. (2020), a single set of bench presses with 80% of one-repetition maximum (1RM) to a 10% decrease in mean velocity, which allowed the participants to perform between 2 and 5 repetitions, lead to a significant acute enhancement in barbell velocity during the subsequent bench press throw. Moreover, it seems that such a volume could even lead to a drop in muscle temperature, not its increase due to reactive vasoconstriction of the skin vessels and redistribution of the blood immediately after a brief bout of intense activity (Merla et al., 2010; Formenti et al., 2016). For example, a slight and non-significant drop in temperature was observed by Weigert et al. (2018) after ten repetitions of biceps curls at 70% 1RM. Therefore, since even a low-volume CA may increase subsequent performance above and beyond that of the warm-up, the involvement of other mechanisms cannot be excluded.

Two crucial factors should be considered to maximize the PAPE effect: an optimal balance between fatigue and potentiation; and similarity in terms of movement pattern between the CA and subsequent performance. Therefore, the CA should induce a high level of potentiation and a low level of fatigue and, simultaneously, imitate the following explosive task in terms of movement structure and the range of motion (Dello Iacono et al., 2016; Krzysztofik et al., 2022). When considering the meaning of fatigue in successfully inducing the PAPE effect, it could be useful to monitor its kinetics following the CA. One of those solutions could include assessing viscoelastic properties, such as tendon stiffness, *via* myotonometry. For example, Pożarowszczyk et al. (2018) reported a CA-induced increase in Achilles tendon stiffness (after single repetitions of progressive back squats at 60%–100% 1RM with 10% steps). However, the authors did not evaluate how it affects subsequent performance. On the other hand, a study by Gago et al. (2014) showed no change in Achilles tendon stiffness despite the PAPE effect being noticed (significant increase in peak twitch and rate of torque development in the plantar flexor). Interestingly reduced tendon stiffness was reported after brief muscle contractions (Kay et al., 2015), with no influence on performance, while in the case of muscles, an increased stiffness was accompanied by acute fatigue (Wang et al., 2016, 2017). This might indicate that a subtle increase in stiffness may help identify the early development of fatigue and performance impairment. A direct influence of CA on acute changes in tendon stiffness and performance has not been established yet and therefore merits further investigation. Considering the above, an appropriately chosen CA should induce a high level of potentiation and low fatigue; thus, no effect on stiffness would be expected.

It seems that isometric as well as plyometric exercises may be a good choice for CA. Plyometric and exercise induce a relatively low level of fatigue compared to high-loaded resistance exercises (Seitz and Haff, 2016) with preferential recruitment of type II motor units (Desmedt and Godaux, 1977). On the other hand, isometric contractions are highly effective in developing joint-specific forces (Lum and Barbosa, 2019); thus, this type of CA could be beneficial in developing strength in particular phases of motor tasks that are difficult to imitate through isotonic resistance exercises. Therefore, a skillful combination of isometric and plyometric exercises could constitute a complementary CA, which in a very specific way might imitate a subsequent compound athletic task and ensure the principle of similarity. However, to the best of the authors’ knowledge, no studies have investigated such a combination as a CA. Nevertheless, the effectiveness of both forms of exercise used separately as the CA has been confirmed in inducing the PAPE effect (Bogdanis et al., 2014; Krzysztofik and Wilk, 2020). Moreover, a combined sequence of these exercises performed alternatively (high-loaded isometric exercise followed immediately by a high-velocity/plyometric exercise) is called contrast training and is effectively used in long-term power development (Cormier et al., 2020).

Bearing in mind the principle of similarity and that most specific sports tasks are based on unilateral movements, the use of bilateral CA seems to be suboptimal. For example, Escobar (Escobar Hincapié et al., 2021) showed a significant decrease in countermovement jump height (CMJ) after both bilateral and split squats (3 sets of 3 repetitions at approximately 87%1RM) as a CA. However, the authors noted an improvement in the change of direction (COD) speed assessed by the t-agility test, with a greater effect after the split squat CA. On the other hand, a study by Lockie et al. (2017) assessed the effectiveness of high-loaded (5 repetitions for each leg at 85% 1RM) dumbbell walking lunges as the CA on 20 m sprints (divided into 0–5 and 0–10 m sections) in strength-trained participants. The authors did not show any significant improvements in particular running sections (from 15 s till 16 min following the CA, with 2 min steps). However, when the best individual rest interval after the CA was considered, there was a 1.98% improvement in the 0–5 m interval, indicating that the PAPE response was greatest during the acceleration phase. This could partially explain the slightly superior effects of unilateral CA in the t-agility test noted by Escobar Hincapié et al. (2021). This test requires multiple changes in running direction over short distances of 5 and 10 m, thus multiple accelerations and decelerations.

Considering the lack of data about the effectiveness of bilateral and unilateral CAs and the combination of maximal intensity isometric and plyometric exercises on the PAPE effect and Achilles tendon stiffness, this study aimed to compare the effects of the two CAs: 1) bilateral isometric half back squats followed by bilateral drop jumps (BI-CA), and 2) unilateral isometric half back squats followed by unilateral drop jumps (UNI-CA) on CMJ performance, modified t-agility test, Achilles tendon stiffness, and skin surface temperature. We hypothesized that both CA would significantly improve CMJ and COD performance; however, the magnitude of improvement would vary and depend on the CA used. Thus, we assumed that the bilateral CA would improve the CMJ performance to a greater magnitude than the unilateral CA, while the unilateral CA would be superior in COD improvement to bilateral CA. In addition, we assumed that both CAs would raise the temperature similarly and would have no effect on Achilles tendon stiffness.

## Methods

### Participants

Thirteen female semi-professional volleyball players (second division in Poland) participated in this study (age: 25 ± 4 years, body mass: 70.9 ± 8.4 kg, body height: 178 ± 4 cm, volleyball training experience: 12 ± 5 years, resistance training experience: 4 ± 2 years, relative one-repetition maximum back squat strength: 1.35 ± 0.17 kg/bm). The inclusion criteria were as follows: 1) free from neuromuscular and musculoskeletal disorders, 2) no lower-limb surgery for 2 years before the study, 3) at least 6 years’ volleyball training and competition experience, 4) regular volleyball and resistance training, and competition for 2 years before the study. Participants were instructed to maintain their usual dietary and sleep habits and not to use stimulants and alcoholic drinks throughout the study. Moreover, they were asked not to perform additional resistance exercises 48-h before testing to avoid fatigue. Participants were allowed to withdraw from the experiment at any time. They were informed about the benefits and potential risks of the study before providing their written informed consent for participation. The participants were not informed about the expected study outcomes. The Bioethics Committee for Scientific Research at the Academy of Physical Education in Katowice, Poland, approved the study protocol. Moreover, it was performed according to the Declaration of Helsinki 2013. The sample size was calculated *a priori* based on a statistical power of 0.8, an effect size of g = 0.46–0.81, and a significance level of 0.05, taking acute changes in stiffness after exercise (Wang et al., 2017), and post-activation performance enhancement in the change of direction performance (Marshall et al., 2019) as a reference variable. A minimum sample size of between 6–12 individuals was obtained (G*Power [version 3.1.9.2], Dusseldorf, Germany).

### Experimental sessions

**FIGURE 1:**
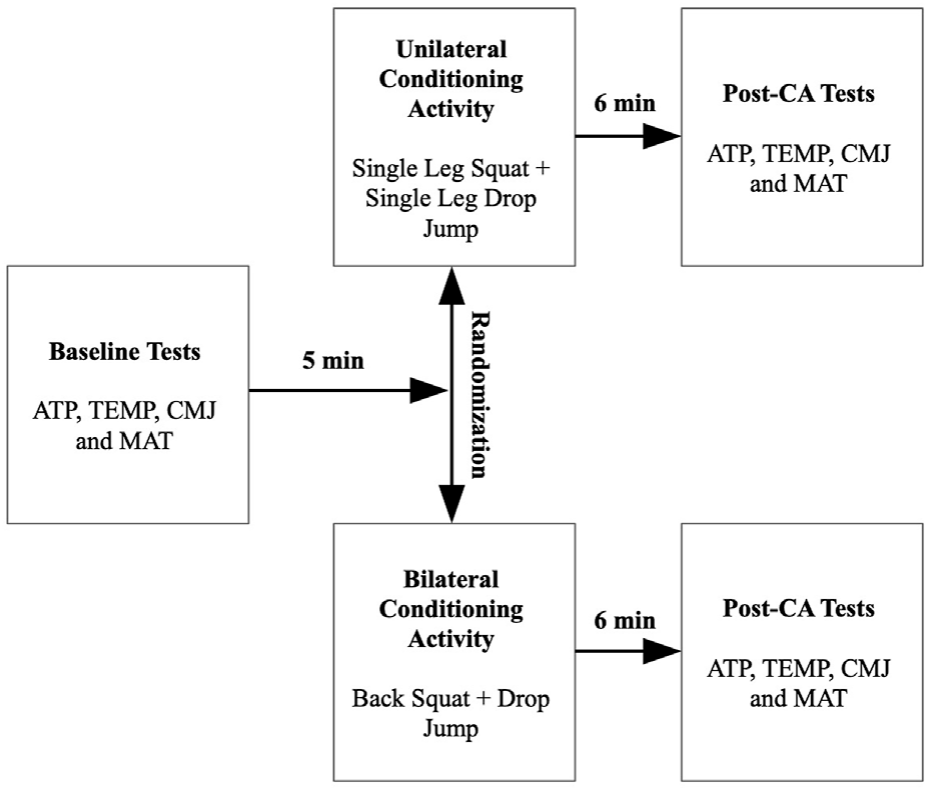
Study design. ATS, Achilles tendon stiffness; TEMP, skin surface temperature; CMJ, countermovement jump; MAT, modified agility T-test; CA, conditioning activity.

The experiment was performed following a randomized crossover design. Each participant performed two experimental sessions to compare the acute effects of unilateral (U-CA) and bilateral (B-CA) on countermovement jump kinematic variables, modified agility T-test, Achilles tendon stiffness, and lower-limbs skin surface temperature. Measurements were performed 5 min before and 6 min after the CA (Figure 1).

#### Conditioning activity

After a standardized warm-up and baseline assessments, the participants performed two different CA in a randomized order: 1) 2 sets of bilateral isometric half back squats followed by bilateral drop jumps (B-CA) and 2) 2 sets of unilateral isometric single leg squats followed by a unilateral drop jump (U-CA). During the squats on the barbell, there was a load significantly exceeding the one-repetition maximum of the participants, which made it impossible to perform the concentric movement. Each participant was asked to adopt a preferred knee flexion corresponding to the knee flexion they obtained during the CMJ. Based on this, the height of the stands was determined. Participants were instructed to push the barbell as forcefully and quickly as possible. To equate the CA volume, in each set, the participants performed: 6 s maximal attempts of bilateral half back squats and then immediately 10 repetitions of drop jumps (from 55 cm height) or 3 s on each leg during unilateral single leg squats and then immediately 5 repetitions of drop jump on each leg. Three-minute rest intervals between sets were adopted.

#### Measurement of jumping performance

Jumping performance was measured using force plates (Force Decks, Vald Performance, Australia). This device has been previously confirmed as a valid and reliable (Heishman et al., 2020) for assessing vertical jump kinematics. Each participant performed two CMJ without arm swing at pre-CA as a baseline and 6 min post-CA. For this measurement, the participant started in the standing position with hands placed on the hips. Next, they dropped into the countermovement position to a self-selected depth, followed by a maximal effort vertical jump. The participants were instructed to land in the same position as the take-off in the mid-section of the force plate. After each jump, the participant reset to the starting position, and the procedure was completed for a total of two jumps. Jump height (JH), reactive strength index modified (RSImod—as a ratio of jump height and contraction time), relative peak power (RPP), relative peak force (RPF), peak velocity (PV), contraction time (CT), and countermovement depth (CD) were evaluated. The best jump in terms of height was kept for further analysis. The stronger lower limb was defined as that which generated a higher relative peak force.

#### Measurement of change of direction time

The modified agility T-test was selected to assess this ability since its replicates the nature of displacements in sports such as the volleyball (Sassi et al., 2009). The participants started the test from a semi-crouched position facing forwards with the front foot placed 0.5 m behind the first timing gate to prevent any early triggering of the start gate. The participants sprinted 5 m forwards to touch the top of the middle cone, then shuffled 2.5 m to the left to touch the next cone, shuffled 5 m in the opposite direction, touched the cone, shuffled 2.5 m back to the middle marker, and finally pedaled back through the timing gates to the finish. Two trials were completed. One min rest intervals were used between attempts. Running times were recorded using Witty Gate timing photocells (Microgate, Bolzano, Italy). The height was set at approximately 1 m off the ground, corresponding to participants’ hip height, to avoid the timing gates being triggered prematurely by a swinging arm or leg. The participants started with the front foot placed 0.3 m behind the timing gate to prevent any early triggering of the photocells. The best running performance was kept for further analysis.

#### Measurement of skin surface temperature

The FLIR E54 infrared camera (FLIR Systems, Inc., United States) was used for thermographic images and then analyzed with FLIR Research Software (FLIR Systems, Inc., United States). The average temperature from the quadriceps muscle zone according to criteria set out by Fernández-Cuevas et al. (2014) was evaluated. The camera was calibrated by a black body; the emissivity was set at 0.97–0.98. Thermal images were made taking into consideration a checklist directed at standardizing thermographic imaging in sports and exercise medicine by Moreira et al. (2017). The participants stood perpendicular, at a distance of 1.5 m from the camera in front of a white uniform background. There was a constant temperature (21°C), the intensity of light, and no direct ventilation in the test room.

#### Measurement of Achilles tendon stiffness

The MyotonPRO hand-held myometer (MyotonPRO, Myoton AS, Tallinn, Estonia) was used for the non-invasive assessment of the Achilles tendon stiffness of both limbs. In the prone position, the measurement was performed 2 cm proximal to the superior aspect of the calcaneus at 0° of the ankle joint dorsiflexion (Taş and Salkın, 2019). The Myoton’s accelerometer was set at 3200 Hz with an average value obtained from five consecutive measurements (0.4 N for 15 ms).

### Statistical analysis

All statistical analyses were performed using SPSS (version 25.0; SPSS, Inc., Chicago, IL, United States) and were shown as means with standard deviations (±SD) with their 95% confidence intervals (CI). Statistical significance was set at *p* < 0.05. The normality of data distribution was checked using Shapiro–Wilk tests. The relative reliability was assessed by intraclass correlation coefficient (two-way mixed effects, absolute agreement, single rater) and absolute reliability with a coefficient of variation. The thresholds for interpreting intraclass correlation coefficient results were: <0.5 “poor”, 0.5–0.75 “moderate”, <0.76–0.9 “good”, and >0.90 as “excellent” (Koo and Li, 2016), while for coefficient of variation the results were: <10% “very good”, 10%–20% “good”, <21–30% “acceptable”, >30% “not acceptable” (Campbell et al., 2021). The two-way ANOVAs (2 × [B-CA; U-CA] × 2 time-points [pre-CA; post-CA]) were used to investigate the influence of CA on MAT time, selected CMJ variables (JH, PV, RPP, CT, CD), and skin surface temperature. The three-way ANOVAs (2 × [B-CA; U-CA] × 2 time-points [pre-CA; post-CA] × 2 limb [stronger; weaker]) were used to determine the influence of the CA on PF during the CMJ and Achilles tendon stiffness. When a significant main effect or interaction was found, the post-hoc tests with Bonferroni’s correction were used to analyze pairwise comparisons. However, when the data normality was not confirmed, related samples Friedman’s two-way ANOVA by ranks were used, and Kendall’s coefficient of concordance estimated the effect size [ES]. The magnitude of mean differences was expressed with standardized ES. Thresholds for qualitative descriptors of Hedges g were interpreted as ≤0.20 “small,” 0.21–0.79 “medium,” and >0.80 as “large.” The smallest worthwhile change (SWC, calculated using formula 0.2 × test-values standard deviation) was used to define the responders and non-responders to the CA. A participant was considered as: 1) a responder if the increase in jump height exceeded the SWC value, 2) a non-responder if jump height change fell within the SWC value, and 3) a negative responder if jump height decreased above SWC.

## Results

The reproducibility of measured data is presented in Table 1.

**TABLE 1:** Intersession reliability of the countermovement jump performance, modified t-test, Achilles tendon stiffness, and skin surface temperature.

Variable	ICC [95%CI]	CV (%)
Jump Height	0.97 (0.9–0.99)	3.6
Relative Peak Power	0.89 (0.67–0.97)	4.7
Peak Velocity	0.89 (0.60–0.96)	2.8
RSImod	0.88 (0.63–0.96)	7.9
Contraction Time	0.93 (0.76–0.98)	6.5
Countermovement Depth	0.88 (0.62–0.96)	4.1
Stronger Limb Relative Peak Force	0.92 (0.73–0.97)	3.4
Weaker Limb Relative Peak Force	0.89 (0.62–0.97)	4.1
Modified T-test	0.96 (0.86–0.99)	1.2
Stronger Limb Achilles Tendon Stiffness	0.92 (0.73–0.97)	3.1
Weaker Limb Achilles Tendon Stiffness	0.88 (0.63–0.96)	4
Skin surface temperature	0.87 (0.61–0.96)	0.7

ICC, intraclass correlation coefficient; CI, confidence intervals; CV, coefficient of variation.

### Jumping performance

The SWC for jump height was 1.1 cm for BI-CA and 1 cm for UNI-CA. During BI-CA, 10 participants were considered as a responder, 2 as negative responders, and one as non-responder. During UNI-CA, 5 participants were considered as responders, 7 as non-responders, and one as a negative responder.

The Shapiro–Wilk tests indicated a violation of jump height and peak velocity data distribution. Friedman’s test showed no differences in peak velocity, but significant differences in jump height (test = 13.881; *p* = 0.003; W = 0.356) were found. Pairwise comparisons demonstrated a significant increase from pre-to post-CA jump height during the BI-CA condition (*p* = 0.005) (Table 2).

**TABLE 2:** Comparisons of pre- and post-CA countermovement jump and modified t-test variables.

Variable	BI-CA				UNI-CA			
	Pre-CA	Post-CA	ES	Δ	Pre-CA	Post-CA	ES	Δ
Jump Height [cm]	32.5 ± 4.9 (29.4–35.4)	34.7 ± 4.6 (31.9–37.4)^a^	0.45	7.7 ± 8.5%	33.2 ± 5.5 (29.9–36.5)	34.2 ± 5.5 (30.9–37.5)	0.18	3.1 ± 6%
Relative Peak Power [W/kg]	46.5 ± 5.8 (43.0–49.0)	48.4 ± 4.9 (45.5–51.4)	0.34	4.7 ± 7.2%	48.2 ± 6.87 (44.0–52.3)	48.0 ± 7.5 (43.5–52.5)	−0.03	−0.4 ± 3.7%
Peak Velocity [m/s]	2.59 ± 0.25 (2.44–2.74)	2.68 ± 0.21 (2.55–2.81)	0.38	3.7 ± 6.2%	2.62 ± 0.17 (2.52–2.72)	2.64 ± 0.22 (2.51–2.78)	0.1	0.8 ± 3.6%
Relative Stronger Limb Peak Force [N/kg]	11.38 ± 1.26^b^	11.67 ± 1.22^b^ ^c^	0.23	2.7 ± 4.8%	11.64 ± 1.05^b^	11.31 ± 1.02^a^ ^b^	−0.31	−2.7 ± 3.6%
Relative Weaker Limb Peak Force [N/kg]	10.82 ± 1.21	11.04 ± 1.19	0.18	2.2 ± 4.4%	11.20 ± 1.13	10.9 ± 1.09^a^	−0.26	−2.6 ± 4.0%
RSImod [m/s]	0.33 ± 0.06	0.36 ± 0.06^a^	0.48	12 ± 15%	0.34 ± 0.07	0.36 ± 0.08	0.26	4 ± 9%
Contraction Time [ms]	1028 ± 255	981 ± 206	−0.2	−2.5 ± 14.4%	1019 ± 291	1008 ± 294	−0.04	0.7 ± 9.3%
Countermovement Depth [cm]	30.7 ± 2.9	30.3 ± 2.2	−0.15	−0.8 ± 7.1%	30.2 ± 3.9	32.4 ± 5.6	0.44	7.5 ± 12.3%
Modified T-test [s]	6.464 ± 0.401 (6.222–6.706)	6.407 ± 0.457 (6.130–6.684)	−0.13	−0.9 ± 3.7%	6.485 ± 0.411 (6.236–6.733)	6.358 ± 0.505 (6.053–6.664)	−0.27	−2.0 ± 3.7%

^a^ Significant difference in comparison to pre-CA value within condition.

^b^ Significant difference in comparison to weaker limb.

^c^ Significant difference in comparison to a corresponding time point in unilateral conditioning activity condition; BI-CA, bilateral conditioning activity; UNI-CA, unilateral conditioning activity; ES, effect size.

The two-way ANOVA did not indicate a significant interaction nor main effects for CD (F = 4.558, *p* = 0.054, η^2^ = 0.275; main effect of time: F = 3.238, *p* = 0.097, η^2^ = 0.212; main effect of condition: F = 0.729, *p* = 0.41, η^2^ = 0.057) and CT (F = 0.407, *p* = 0.536, η^2^ = 0.033; main effect of time: F = 1.647, *p* = 0.224, η^2^ = 0.121; main effect of condition: F = 0.062, *p* = 0.807, η^2^ = 0.005) (Table 2).

The two-way ANOVA indicated a significant main effect of time (F = 17.877, *p* = 0.001, η^2^ = 0.598) for RSImod. The post-hoc comparison showed a significant increase of RSImod from pre-to post-CA during the BI-CA condition (*p* = 0.008).

The three-way ANOVA indicated a significant condition × time interaction (F = 10.226, *p* = 0.008, η^2^ = 0.460) and a main effect of limb (F = 20.299, *p* = 0.001, η^2^ = 0.628) CMJ relative peak force. The post-hoc comparisons showed a significantly higher, stronger limb CMJ relative peak force in BI-CA compared to UNI-CA at post-CA (*p* = 0.005; ES = 0.31). Moreover, stronger and weaker limb CMJ relative peak force significantly decreased from pre-to post-CA (*p* = 0.014 and *p* = 0.027; respectively) during UNI-CA. In addition, the stronger limb’s relative CMJ peak force was higher in comparison to the weaker limb at all time points (*p* < 0.009; ES = 0.38–0.51) in both conditions.

### Change of direction time

The two-way ANOVA did not indicate a significant interaction (F = 0.994, *p* = 0.338, η^2^ = 0.076) nor main effect of time (F = 2.788, *p* = 0.121, η^2^ = 0.189) and condition (F = 0.055, *p* = 0.819, η^2^ = 0.005) for MAT time (Table 2).

### Skin surface temperature

**FIGURE 2:**
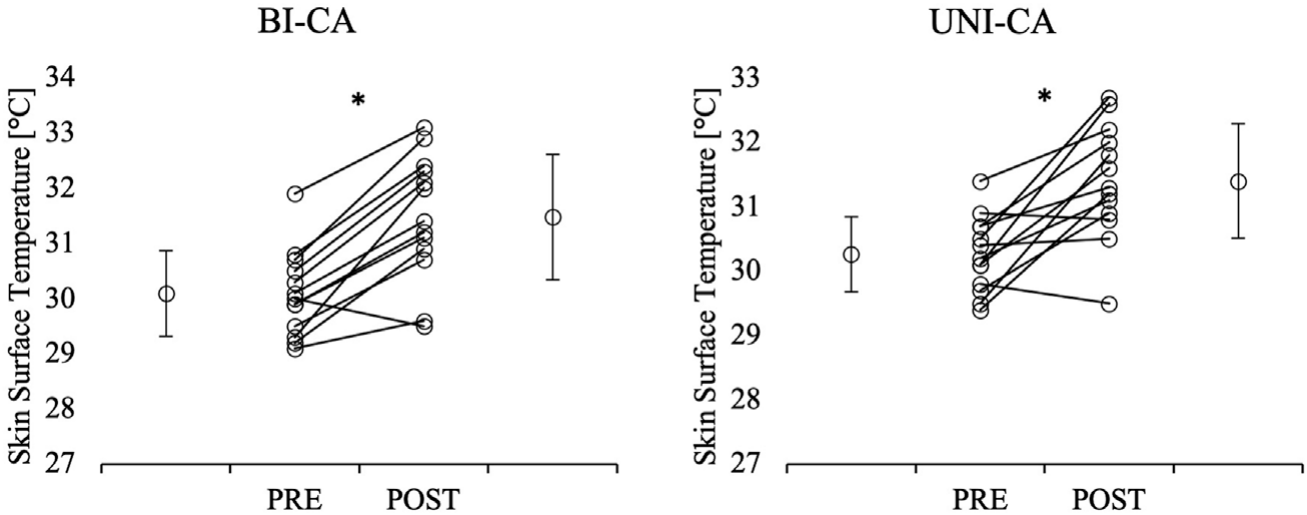
Comparisons of pre- and post-CA quadriceps muscles skin surface temperature. *significant difference in comparison to pre-CA; BI-CA, bilateral conditioning activity; UNI-CA, unilateral conditioning activity.

The two-way ANOVA indicated a significant main effect of time (F = 82.188, *p* < 0.001, η^2^ = 0.873) for temperature. The post-hoc comparison showed a significant increase in temperature from pre- to post-CA during BI-CA (*p* < 0.001) and UNI-CA (*p* = 0.001) conditions (Figure 2).

### Achilles tendon stiffness

**FIGURE 3:**
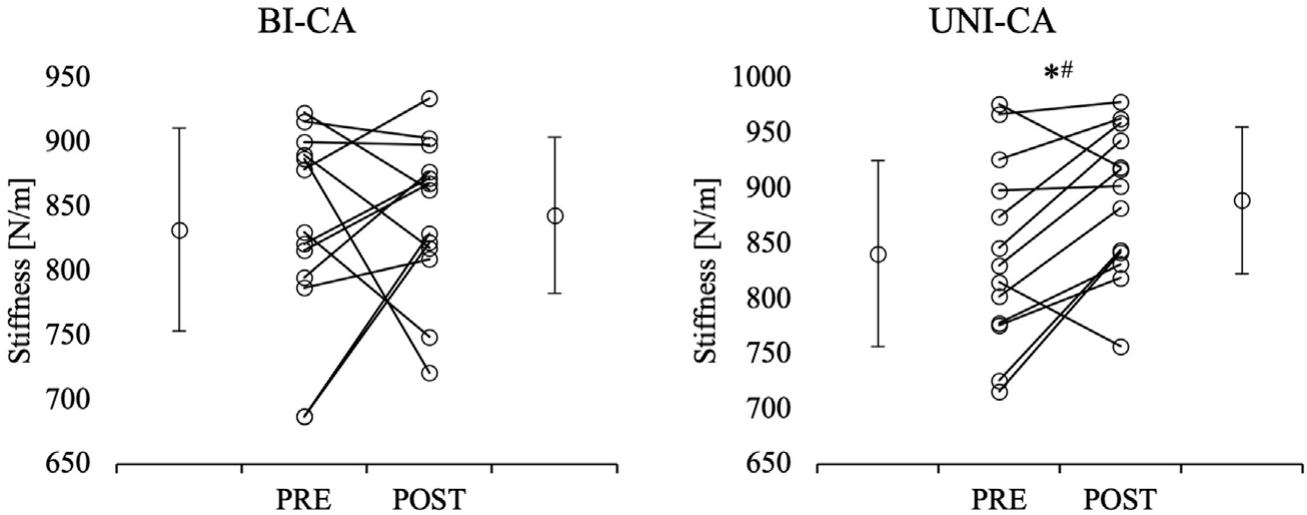
Comparisons of pre- and post-CA Achilles tendon stiffness in stronger limb. *significant difference in comparison to pre-CA value within condition; °significant difference in comparison to corresponding time point in bilateral conditioning activity condition; BI-CA, bilateral conditioning activity; UNI-CA, unilateral conditioning activity.

**FIGURE 4:**
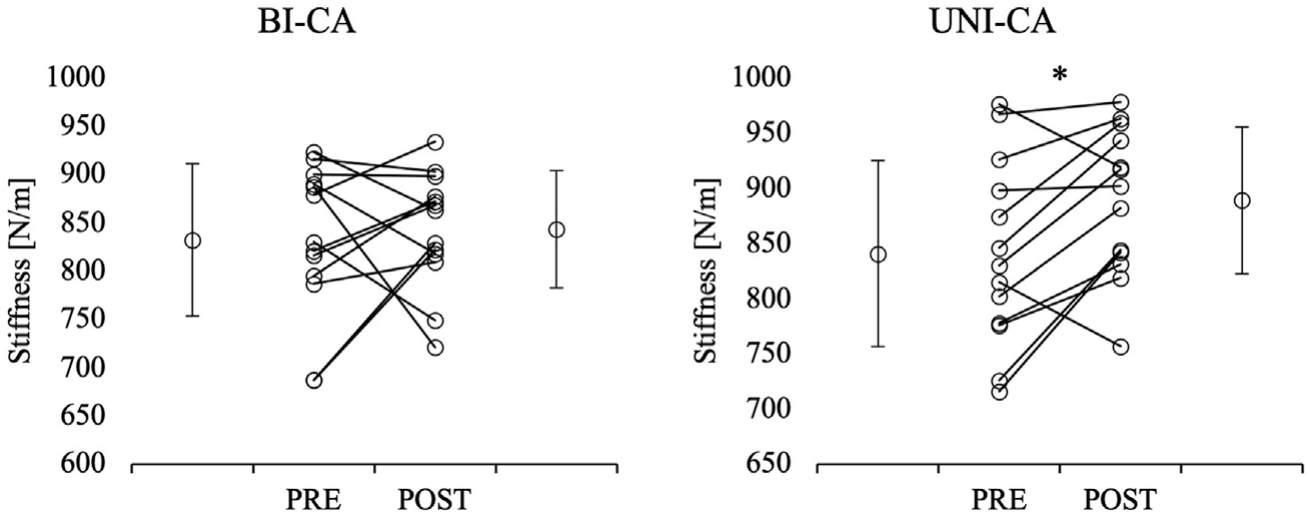
Comparisons of pre- and post-CA Achilles tendon stiffness in weaker limb. *significant difference in comparison to pre-CA value within condition; BI-CA, bilateral conditioning activity; UNI-CA, unilateral conditioning activity.

The three-way ANOVA indicated a significant main effect of condition (F = 8.908, *p* = 0.011, η^2^ = 0.426) for Achilles tendon stiffness. No other interactions or main effects were reported. The post-hoc comparison showed a significant increase in stronger and weaker limb Achilles tendon stiffness from pre-to post-CA during the UNI-CA condition (*p* = 0.013 and *p* < 0.001, respectively). Moreover, the post-CA stronger limb Achilles tendon stiffness during the UNI-CA was significantly higher in comparison to post-CA stronger limb Achilles tendon stiffness during the BI-CA (*p* = 0.006; ES = 0.7) (Figures 3, 4).

## Discussion

The main finding of this study was that the bilateral CA significantly increases CMJ height and RSImod compared to the unilateral CA among female volleyball players. In addition, none of the used CAs improved the MAT time test and affected contraction time and countermovement depth during CMJ. Moreover, the CMJ relative peak force of both limbs significantly decreased after UNI-CA with a concomitant increase in Achilles tendon stiffness. Both conditions significantly increased lower-limb skin surface temperature. These findings support the principle of similarity between the CA and the subsequent explosive task to induce the PAPE effect.

The assumed hypothesis was partially confirmed as only the BI-CA contributed to a significant improvement in CMJ performance, and neither CA improved COD performance. These findings are in contrast to the results of a study by Escobar (Escobar Hincapié et al., 2021), which showed a significant decrease in CMJ height performed 5 min after a CA consisting of 3 sets of 3 repetitions of bilateral or split squats at 0.59 m/s (approximately equivalent to 87% 1RM). However, both CAs significantly improved COD performance assessed by the t-agility test, with a slightly greater effect after the split squat CA. These differences could be due to a short rest interval after the CA. Nevertheless, this was not confirmed by the fact that the PAPE effect was observed in the COD test. Thus, the reasons for these differences between the Escobar (Hincapié et al., 2021) study and the current one is difficult to explain.

The lack of CMJ improvements in our research may be explained by the induction of a higher level of fatigue in the UNI-CA compared to BI-CA. Despite the unification of exercise volume between the CAs (in both, 10 repetitions in total were performed), the intensity of each individual unilateral jump is higher as it involves the same amount of load (bodyweight) but is performed on a single limb. Hence, the isometric split squat and the single-leg drop jump caused greater fatigue than the bilateral combination. This can be confirmed by a significant decrease in the generated force during the CMJ and a significant increase in Achilles tendon stiffness. Consistent with our findings, recent work has identified increased stiffness in the patellar tendon and a decrease in maximum voluntary isometric contraction following a single bout of maximal eccentric knee extensions (Heales et al., 2018). This seems likely because unilateral exercise can cause more damage and fatigue than its bilateral counterparts (Costa et al., 2015; Isik and Dogan, 2018).

However, compared to previous studies (Heales et al., 2018), the increase in stiffness was not accompanied by a significant decrease in CMJ height. Taking into account the importance of the relation between fatigue and potentiation in the effective induction of the PAPE effect, it seems that the level of fatigue was balanced with the induced potentiation; hence the performance was not influenced. On the other hand, although not significant, numerically, the change in CD from pre-to post-CA during UNI-CA was greater (ES = 0.44; Δ = 7.5%) compared with the BI-CA (ES = −0.15; Δ = -0.8%). Therefore, this might indicate that due to fatigue, the participants changed the jumping strategy by performing a slightly deeper countermovement which was accompanied by a lower relative peak force and no significant change in jump height within the UNI-CA condition (Sánchez-Sixto et al., 2018; Bishop et al., 2022). Nevertheless, one of our study’s limitations was that we examined performance only at a single time-point, 6 min after the CA. Consequently, it cannot be ruled out that the performance enhancement after the UNI-CA could have occurred later.

It is also possible that the lack of significant improvement in the CMJ after the UNI-CA is due to the principle of exercise similarity in PAPE protocols. A significant increase was observed only after the BI-CA. Both CAs used a knee flexion similar to that obtained during the CMJ; however, the BI-CA, in a closer way, mimics the CMJ. At the same time, it is worth noticing that despite the lack of significance, a slightly greater improvement was registered in MAT after the UNI-CA. This may be justified by a more remarkable similarity of the split squat to the running gait structure (Lockie et al., 2017). Nevertheless, the COD test used in this study consists mainly of lateral movements; thus, the UNI-CA used could not reflect the involvement of the same muscle groups. The importance of exercise similarity has been pointed out in previous studies (Dello Iacono et al., 2016; Krzysztofik et al., 2022). For example, Krzysztofik et al. (2022) showed that the CA and subsequent explosive task should be as similar as possible regarding the range of motion to maximize the PAPE effect. In another study, Dello Iacono et al. (2016) compared the effects of alternating single-leg drop jumps performed horizontally and vertically (3 × 5 each leg, from a 25 cm height) as a CA on CMJ, 10 m sprint, and t-agility test performed 8 min later. The results indicated a specific effect of the CA; the horizontal drop jumps enhanced the t-agility test while vertical drop jumps improved the CMJ performance. A significant increase in RSImod without an improvement in MAT found in this study may also confirm this finding. A high level of reactive force is essential in high-intensity motor tasks such as acceleration, deceleration, or changing directions (Alenezi et al., 2014). However, the improvement in RSImod was only accompanied by a significant improvement in CMJ, and the one registered in the MAT was insignificant. This may be related to the fact that RSImod in this study was related to vertically generated explosive force, not horizontal or lateral force; thus, the most likely reason it did not translate into improvement in MAT. However, if a different COD assessment test were used in the procedure without lateral movements (e.g., the 5-0-5 test), perhaps the observed improvement would exceed the significance level. Therefore, it seems that a combination of an isometric split squat and, e.g., lateral shuffles could be a suitable CA as an alternative to enhance performance in this COD test. Therefore, we suggest that future studies evaluate different exercise combinations as CAs to improve performance in complex motor tasks.

To the best of the authors’ knowledge, this is the first study to assess skin surface temperature for standardization of warm-up procedures before the PAPE protocol. For both of the CAs used, a similar increase in surface temperature of the lower limbs was noted, even though only BI-CA improved the CMJ performance. This shows that while the temperature certainly impacts the physical fitness (Racinais et al., 2017), its part may not be relevant in the case of the PAPE effect. Nevertheless, additional research is needed to confirm this speculation.

As highlighted earlier, some limitations should be considered when interpreting this study’s results. We enrolled amateur volleyball female athletes with low relative strength levels; therefore, caution is needed when extrapolating these results to alternative populations and conditions. In addition, we did not measure the ground reaction forces during the CAs; thus, despite the unification of exercise volume, they could represent a different stimulus in terms of induced fatigue and potentiation. Besides, we did not assess the unilateral post-CA performance. Moreover, we measured only Achilles tendon stiffness. Therefore, the lack of influence of the applied CAs on the changes in the muscles of the thighs and hips cannot be ruled out, taking into account that those muscles are highly involved during squats and jumps. Furthermore, only a single rest interval was applied for all participants; therefore, it is possible that for some of them, the PAPE response would occur at different time points post-CA.

## Conclusion

The present study showed that combined isometric and plyometric bilateral CA effectively improved the CMJ but did not enhance the t-agility test performance. These findings indicate that exercise combinations could be effectively used to produce a PAPE effect but should replicate the following explosive task as much as possible. These findings offer important new insights into using PAPE protocols in the practical field and indicate the need to test different combinations of exercises as CA to improve the following complex athletic tasks.

## Data Availability

The raw data supporting the conclusions of this article will be made available by the authors, without undue reservation.

## References

[B1] Alenezi F. Herrington L. Jones P. Jones R. (2014). Relationships between lower limb biomechanics during single leg squat with running and cutting tasks: A preliminary investigation. Br. J. Sports Med. 48, 560.3–561. 10.1136/bjsports-2014-093494.3

[B2] Bishop C. Turner A. Jordan M. Harry J. Loturco I. Lake J. (2022). A framework to guide practitioners for selecting metrics during the countermovement and drop jump tests. Strength Cond. J. 44, 95–103. 10.1519/SSC.0000000000000677

[B3] Blazevich A. J. Babault N. (2019). Post-activation potentiation versus post-activation performance enhancement in humans: Historical perspective, underlying mechanisms, and current issues. Front. Physiol. 10, 1359. 10.3389/fphys.2019.01359 31736781 PMC6838751

[B4] Bogdanis G. C. Tsoukos A. Veligekas P. Tsolakis C. Terzis G. (2014). Effects of muscle action type with equal impulse of conditioning activity on postactivation potentiation. J. Strength Cond. Res. 28, 2521–2528. 10.1519/JSC.0000000000000444 24584048

[B5] Campbell M. J. Walters S. J. K. Machin D. (2021). Medical statistics: A textbook for the health sciences. Fifth Edition. Hoboken, NJ: John Wiley Blackwell.

[B6] Chudecka M. (2013). The use of thermal imaging in the evaluation of the body surface temperature in various physiological states and in patients with different body composition and varying levels of physical activity. Cent. Eur. J. Sport Sci. Med. 2, 15–20.

[B7] Cormier P. Freitas T. T. Rubio-Arias J. Á. Alcaraz P. E. (2020). Complex and contrast training: Does strength and power training sequence affect performance-based adaptations in team sports? A systematic review and meta-analysis. J. Strength Cond. Res. 34, 1461–1479. 10.1519/JSC.0000000000003493 32084104

[B8] Costa E. Moreira A. Cavalcanti B. Krinski K. Aoki M. (2015). Effect of unilateral and bilateral resistance exercise on maximal voluntary strength, total volume of load lifted, and perceptual and metabolic responses. Biol. Sport 32, 35–40. 10.5604/20831862.1126326 25729148 PMC4314602

[B9] Costello J. Stewart I. Selfe J. Karki A. Donnelly A. (2013). The use of thermal imaging in sports medicine research: A short report. Int. SportMed J. 14, 94–98.

[B10] Dello Iacono A. Martone D. Padulo J. (2016). Acute effects of drop-jump protocols on explosive performances of elite handball players. J. Strength Cond. Res. 30, 3122–3133. 10.1519/JSC.0000000000001393 26958786

[B11] Desmedt J. E. Godaux E. (1977). Ballistic contractions in man: Characteristic recruitment pattern of single motor units of the tibialis anterior muscle. J. Physiol. 264, 673–693. 10.1113/jphysiol.1977.sp011689 845820 PMC1307786

[B12] Escobar Hincapié A. Agudelo Velásquez C. A. Ortiz Uribe M. García Torres C. A. Rojas Jaramillo A. (2021). Unilateral and bilateral post-activation performance enhancement on jump performance and agility. Int. J. Environ. Res. Public Health 18, 10154. 10.3390/ijerph181910154 34639455 PMC8508041

[B13] Fernández-Cuevas I. Sillero-Quintana M. Garcia-Concepcion M. A. Ribot Serrano J. Gomez-Carmona P. Cb Marins J. (2014). Monitoring skin thermal response to training with infrared thermography. New Stud. Athl. 29, 57–71.

[B14] Formenti D. Ludwig N. Trecroci A. Gargano M. Michielon G. Caumo A. (2016). Dynamics of thermographic skin temperature response during squat exercise at two different speeds. J. Therm. Biol. 59, 58–63. 10.1016/j.jtherbio.2016.04.013 27264889

[B15] Gago P. Arndt A. Tarassova O. Ekblom M. M. (2014). Post activation potentiation can be induced without impairing tendon stiffness. Eur. J. Appl. Physiol. 114, 2299–2308. 10.1007/s00421-014-2945-3 25048072

[B16] Heales L. J. Badya R. Ziegenfuss B. Hug F. Coombes J. S. van den Hoorn W. (2018). Shear-wave velocity of the patellar tendon and quadriceps muscle is increased immediately after maximal eccentric exercise. Eur. J. Appl. Physiol. 118, 1715–1724. 10.1007/s00421-018-3903-2 29855790

[B17] Heishman A. D. Daub B. D. Miller R. M. Freitas E. D. S. Frantz B. A. Bemben M. G. (2020). Countermovement jump reliability performed with and without an arm swing in NCAA division 1 intercollegiate basketball players. J. Strength Cond. Res. 34, 546–558. 10.1519/JSC.0000000000002812 30138237

[B18] Isik O. Dogan I. (2018). Effects of bilateral or unilateral lower body resistance exercises on markers of skeletal muscle damage. Biomed. J. 41, 364–368. 10.1016/j.bj.2018.10.003 30709578 PMC6361852

[B19] Kay A. D. Husbands-Beasley J. Blazevich A. J. (2015). Effects of contract–relax, static stretching, and isometric contractions on muscle–tendon mechanics. Med. Sci. Sports Exerc. 47, 2181–2190. 10.1249/MSS.0000000000000632 25668401

[B20] Koo T. K. Li M. Y. (2016). A guideline of selecting and reporting intraclass correlation coefficients for reliability research. J. Chiropr. Med. 15, 155–163. 10.1016/j.jcm.2016.02.012 27330520 PMC4913118

[B21] Krzysztofik M. Trybulski R. Trąbka B. Perenc D. Łuszcz K. Zajac A. (2022). The impact of resistance exercise range of motion on the magnitude of upper-body post-activation performance enhancement. BMC Sports Sci. Med. Rehabil. 14, 123. 10.1186/s13102-022-00519-w 35799185 PMC9264649

[B22] Krzysztofik M. Wilk M. Stastny P. Golas A. (2021). Post-activation performance enhancement in the bench press throw: A systematic review and meta-analysis. Front. Physiol. 11, 598628. 10.3389/fphys.2020.598628 33519506 PMC7844331

[B23] Krzysztofik M. Wilk M. (2020). The effects of plyometric conditioning on post-activation bench press performance. J. Hum. Kinet. 74, 99–108. 10.2478/hukin-2020-0017 33312279 PMC7706649

[B24] Lockie R. G. Lazar A. Risso F. G. Giuliano D. V. Liu T. M. Stage A. A. (2017). Limited post-activation potentiation effects provided by the walking lunge on sprint acceleration: A preliminary analysis. Open Sports Sci. J. 10, 97–106. 10.2174/1875399X01710010097

[B25] Lum D. Barbosa T. M. (2019). Brief review: Effects of isometric strength training on strength and dynamic performance. Int. J. Sports Med. 40, 363–375. 10.1055/a-0863-4539 30943568

[B26] Marshall J. Turner A. N. Jarvis P. T. Maloney S. J. Cree J. A. Bishop C. J. (2019). Postactivation potentiation and change of direction speed in elite Academy rugby players. J. Strength Cond. Res. 33, 1551–1556. 10.1519/JSC.0000000000001834 28166184

[B27] Merla A. Mattei P. A. Di Donato L. Romani G. L. (2010). Thermal imaging of cutaneous temperature modifications in runners during graded exercise. Ann. Biomed. Eng. 38, 158–163. 10.1007/s10439-009-9809-8 19798579

[B28] Moreira D. G. Costello J. T. Brito C. J. Adamczyk J. G. Ammer K. Bach A. J. E. (2017). Thermographic imaging in sports and exercise medicine: A delphi study and consensus statement on the measurement of human skin temperature. J. Therm. Biol. 69, 155–162. 10.1016/j.jtherbio.2017.07.006 29037377

[B29] Pożarowszczyk B. Gołaś A. Chen A. Zając A. Kawczyński A. (2018). The impact of post activation potentiation on Achilles tendon stiffness, elasticity and thickness among basketball players. Sports 6, 117. 10.3390/sports6040117 PMC6315499 30321992

[B30] Racinais S. Cocking S. Périard J. D. (2017). Sports and environmental temperature: From warming-up to heating-up. Temperature 4, 227–257. 10.1080/23328940.2017.1356427 PMC5605167 28944269

[B31] Sánchez-Sixto A. Harrison A. Floría P. (2018). Larger countermovement increases the jump height of countermovement jump. Sports 6, 131. 10.3390/sports6040131 PMC6316300 30373113

[B32] Sassi R. H. Dardouri W. Yahmed M. H. Gmada N. Mahfoudhi M. E. Gharbi Z. (2009). Relative and absolute reliability of a modified agility T-test and its relationship with vertical jump and straight sprint. J. Strength Cond. Res. 23, 1644–1651. 10.1519/JSC.0b013e3181b425d2 19675502

[B33] Seitz L. B. Haff G. G. (2016). Factors modulating post-activation potentiation of jump, sprint, throw, and upper-body ballistic performances: A systematic review with meta-analysis. Sports Med. 46, 231–240. 10.1007/s40279-015-0415-7 26508319

[B34] Taş S. Salkın Y. (2019). An investigation of the sex-related differences in the stiffness of the Achilles tendon and gastrocnemius muscle: Inter-observer reliability and inter-day repeatability and the effect of ankle joint motion. Foot 41, 44–50. 10.1016/j.foot.2019.09.003 31704588

[B35] Tsoukos A. Brown L. E. Terzis G. Veligekas P. Bogdanis G. C. (2020). Potentiation of bench press throw performance using a heavy load and velocity-based repetition control. J. Strength Cond. Res. 35, S72–S79. 10.1519/JSC.0000000000003633 32398633

[B36] Wang D. De Vito G. Ditroilo M. Delahunt E. (2017). Different effect of local and general fatigue on knee joint stiffness. Med. Sci. Sports Exerc. 49, 173–182. 10.1249/MSS.0000000000001086 27580153

[B37] Wang D. De Vito G. Ditroilo M. Delahunt E. (2016). Effect of sex and fatigue on muscle stiffness and musculoarticular stiffness of the knee joint in a young active population. J. Sports Sci. 35, 1582–1591. 10.1080/02640414.2016.1225973 27590889

[B38] Weigert M. Nitzsche N. Kunert F. Lösch C. Baumgärtel L. Schulz H. (2018). Acute exercise-associated skin surface temperature changes after resistance training with different exercise intensities. Int. J. Kinesiol. Sports Sci. 6, 12. 10.7575/aiac.ijkss.v.6n.1p.12

